# A comprehensive method for determining cellular uptake of purine nucleoside phosphorylase and adenylosuccinate synthetase inhibitors by *H. pylori*

**DOI:** 10.1007/s00253-021-11510-9

**Published:** 2021-09-25

**Authors:** Marta Ilona Wojtyś, Radosław Jaźwiec, Saša Kazazić, Ivana Leščić Ašler, Petar Knežević, Verica Aleksić Sabo, Marija Luić, Elżbieta Katarzyna Jagusztyn-Krynicka, Agnieszka Bzowska

**Affiliations:** 1 Division of Biophysics, Institute of Experimental Physics, Faculty of Physics, University of Warsaw, Pasteura 5, 02-093 Warsaw, Poland; 2 Department of Bacterial Genetics, Institute of Microbiology, Faculty of Biology, University of Warsaw, Miecznikowa 1, 02-096 Warsaw, Poland; 3 Institute of Biochemistry and Biophysics, Polish Academy of Sciences, Pawińskiego 5A, 02-106 Warsaw, Poland; 4 Division of Physical Chemistry, Ruđer Bošković Institute, Bijenička cesta 54, POB 180, 10002 Zagreb, Croatia; 5 Department of Biology and Ecology, Faculty of Sciences, University of Novi Sad, Trg Dositeja Obradovića 2, IV-14, 21000 Novi Sad, Republic of Serbia

**Keywords:** *Helicobacter pylori*, Cells penetration by drug candidates, Salvage pathway enzymes, Formycin, Hadacidin

## Abstract

**Abstract:**

Due to the growing number of *Helicobacter pylori* strains resistant to currently available antibiotics, there is an urgent need to design new drugs utilizing different molecular mechanisms than those that have been used up to now. Enzymes of the purine salvage pathway are possible targets of such new antibiotics because *H. pylori* is not able to synthetize purine nucleotides de novo. The bacterium’s recovery of purines and purine nucleotides from the environment is the only source of these essential DNA and RNA building blocks. We have identified formycins and hadacidin as potent inhibitors of purine nucleoside phosphorylase (PNP) and adenylosuccinate synthetase (AdSS) from *H. pylori* — two key enzymes of the purine salvage pathway. However, we have found that these compounds are not effective in *H. pylori* cell cultures. To address this issue, we have developed a universal comprehensive method for assessing *H. pylori* cell penetration by drug candidates, with three alternative detection assays. These include liquid chromatography tandem mass spectrometry, UV absorption, and inhibition of the target enzyme by the tested compound. Using this approach, we have shown that cellular uptake by *H. pylori* of formycins and hadacidin is very poor, which reveals why their in vitro inhibition of PNP and AdSS and their effect on *H. pylori* cell cultures are so different. The cell penetration assessment method developed here will be extremely useful for validating the cellular uptake of other drug candidates, facilitating the design of new potent therapeutic agents against *H. pylori*.

**Key points:**

• *A method for assessing H. pylori cells penetration by drug candidates is described*.

• *Three alternative detection assays that complement each other can be used*.

• *The method may be adapted for other bacteria as well*.

## Introduction

The gram-negative microaerophilic bacterium *Helicobacter pylori* is a common pathogen affecting about 50% of the human population worldwide (Kamboj et al. 2017). Since its identification by Marshall and Warren (1984), *H. pylori* is seen as a serious threat to human health, through its involvement in the development of diseases such as chronic active gastritis, peptic ulceration, gastric adenocarcinoma, and gastric mucosa-associated lymphoid tissue lymphoma (Abadi 2017). In 1994 it was classified as a class I human carcinogen (Humans 1994; Miftahussurur et al. 2017).

Various therapies are currently used and recommended for eradication of *H. pylori*. The fact that there are so many of them is because none of them is universal and effective in all circumstances and in every region of the world. It is estimated that treatment for *H. pylori* fails in more than 20% of patients (Roszczenko-Jasińska et al. 2020). One of the most popular is a standard triple 10–14 days therapy consisting of a proton pump inhibitor, amoxicillin and clarithromycin or metronidazole (Suzuki and Mori 2018). However, with the growing resistance of *H. pylori* to antimicrobial agents, especially to clarithromycin and metronidazole, in the triple therapy, these antibiotics are often replaced by other drugs, e.g., by levofloxacin, sitafloxacin, or rifabutin (Thung et al. 2016; Suzuki and Mori 2018). Unfortunately, even with these modifications, standard triple therapy is no longer effective in most countries (Huang et al. 2017). Alternative approaches were therefore proposed including high-dose dual, bismuth quadruple and non-bismuth quadruple therapies, the latter one with several variants like sequential, concomitant, hybrid, and reverse hybrid. High-dose dual therapy consists of high-dose proton pump inhibitor and amoxicillin, as they are administrated for 14 days but four times per day (Huang et al. 2017). The 2016 Maastricht V/Florence Consensus Report (Malfertheiner et al. 2017) and the 2016 Toronto Consensus (Fallone et al. 2016) recommended a bismuth quadruple therapy as a first-line choice, especially when resistance to clarithromycin occurs. This treatment includes a proton pump inhibitor, bismuth, metronidazole, and tetracycline; is well tolerated by patients; and shows effectiveness even against in vitro metronidazole-resistant strains. If bismuth is not available, a concomitant quadruple therapy may be used, consisting of a proton pump inhibitor, amoxicillin, metronidazole, and clarithromycin (Kamboj et al. 2017; Fallone et al. 2019; Matsumoto et al. 2019; Suzuki et al. 2019). In a sequential therapy, a proton pump inhibitor and amoxicillin is administrated for the first half of the treatment (5 days), with metronidazole or clarithromycin replacing amoxicillin for the second half (Kamboj et al. 2017). In the hybrid or reverse hybrid therapy, a proton pump inhibitor and amoxicillin are administrated for 10–14 days, while metronidazole and clarithromycin only for the first and the second half, respectively (Huang et al. 2017).

However, given the generally increased use of antibiotics, the burden of resistant strains of *H. pylori* is also expected to rise (Kuo et al. 2021; Megraud et al., 2021). Therefore, the search for novel targets for drugs against *H. pylori*, with different mechanisms of action, is of utmost importance (Roszczenko-Jasińska et al. 2020). High effort in this area resulted in various novel therapeutic regiments that should improve eradication rate achieved by standard therapies and should restrict the increase of antibiotic-resistant bacteria. These are for example probiotics, plant extracts, or inhibitors of biofilm formation (Roszczenko-Jasińska et al. 2020). Nevertheless, new approaches that can serve as a replacement for current therapies are necessary. When the genome of *H. pylori* was published (Tomb et al. 1997), it opened possibilities for studying the physiology of this pathogen (Doig et al. 1999) in order to identify promising new drug targets. Analysis showed that many redundant metabolic pathways are missing in this bacterium, among them the pathway of de novo purine nucleotides synthesis (Hazell and Mendz 1997). Therefore, *H. pylori* relies exclusively on the purine nucleotide cycle, the so-called purine salvage pathway, for acquisition of purine nucleotides, which are the indispensable building blocks of DNA and RNA. This prompted us to study proteins involved in the purine salvage pathway of this bacterium as possible targets for new antibiotics, as has been done with parasitic protozoa (El Kouni 2003), e.g., the *Plasmodium* responsible for malaria (e.g., Ducati et al. 2013).

**Fig. 1:**
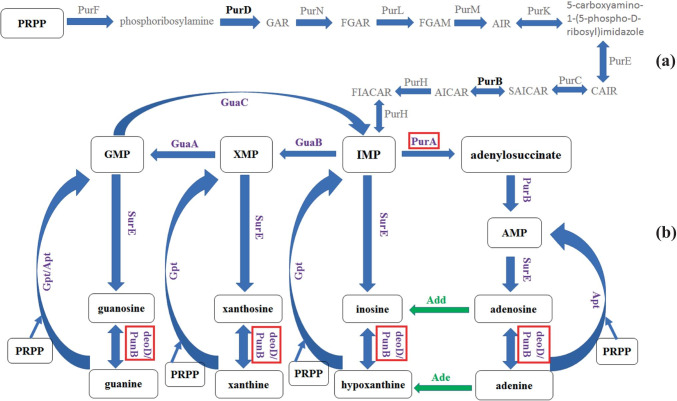
The purine nucleotide biosynthesis pathway in *H. pylori*. (a) De novo purine nucleotide biosynthetic pathway functioning in *E. coli*. Homologs for genes required for this pathway found in *H. pylori* are shown in black, while all enzymes for which homologs were not found in the *H. pylori* genome are marked in gray. (b) Purine salvage pathway in *H. pylori*; purine rings, shown in the bottom row, are obtained from environment. Enzymes that have been studied in *H. pylori* by mutant analysis and/or biochemistry are shown in violet. Enzymes described in this work, purine nucleoside phosphorylase (PunB, PNP) and adenylosuccinate synthetase (PurA, AdSS), are shown in red frames. Enzymes with likely function, but whose genes have not yet been identified are shown in green (figure adapted from Jenkins et al. 2011, Liechti and Goldberg 2012, Miller et al. 2012). Abbreviations: PRPP, 5-phosphoribosyl diphosphate; GAR, glycinamide ribonucleotide; FGAR, N-formylglycinamide ribonucleotide; FGAM, 5′-phosphoribosylformylglycinamidine; AIR, aminoimidazole ribotide; CAIR, 5′-phosphoribosyl-4-carboxy-5-aminoimidazole; SAICAR, 5′-phosphoribosyl-4-(N-succinocarboxamide)-5-aminoimidazole; AICAR, 5-aminoimidazole-4-carboxamide ribotide; FIACAR, 5′-phosphoribosyl-5-formamido-4-imidazolecarboxamide; PurF, amidophosphoribosyltransferase; PurD, phosphoribosylamine-glycine ligase; PurN, phosphoribosylglycinamide formyltransferase; PurL, phosphoribosylformylglycinamidine synthase; PurM, phosphoribosylformylglycinamidine cyclo-ligase; PurK, N5-carboxyaminoimidazole ribonucleotide synthase; PurE, N5-carboxyaminoimidazole ribonucleotide mutase; PurC, phosphoribosylaminoimidazole-succinocarboxamide synthase; PurH, bifunctional purine biosynthesis protein PurH; GuaB, IMP dehydrogenase; GuaA, GMP synthetase; GuaC, GMP reductase; PurA, adenylosuccinate synthetase; PurB, adenylosuccinate lyase; Gpt, hypoxanthine–guanine phosphoribosyl-transferase; Apt, adenine phosphoribosyltransferase; SurE, 5′-nucleotidase; PunB (deoD gene), purine nucleoside phosphorylase; Ade, adenine deaminase; Add, adenosine deaminase; IMP, inosine monophosphate; XMP, xanthosine monophosphate; GMP, guanosine monophosphate; AMP, adenosine monophosphate

Recently published results of Dziekan et al. (2019) have brought additional proof that enzymes of purine salvage pathway may be promising drug targets. They studied quinine and mefloquine, two important antimalarial drugs with poorly characterized mechanisms of action. Using the cellular thermal shift assay coupled with mass spectrometry, they showed that in *Plasmodium falciparum*, purine nucleoside phosphorylase (PNP, E.C. 2.4.2.1) is a common binding target for these two quinoline drugs. Additionally employing biophysical structural studies, the authors have shown that both compounds bind within the enzyme’s active site. Moreover, in solution studies, it was demonstrated that quinine binds to *P. falciparum* PNP with low nanomolar affinity, suggesting a significant role of this interaction in the therapeutic effect of the drug. PNP is a key enzyme of the purine salvage pathway (Fig. 1b), as it catalyzes the reversible phosphorolytic cleavage of the glycosidic bond of purine ribo- and deoxyribonucleosides, inosine, adenosine, and guanosine, as follows: (deoxy)purine nucleoside + orthophosphate → purine base + (deoxy)ribose-1-phosphate.

Using the well-characterized *Escherichia coli* purine nucleoside biosynthesis pathway as a template, Liechti and Goldberg (2012) generated mutants of *H. pylori* with deletions of genes for numerous putative purine nucleoside salvage pathway elements — among them *deoD* and *purA* genes encoding for PNP and adenylosuccinate synthetase (AdSS), respectively. AdSS (E.C. 6.3.4.4) catalyzes the first step in the biosynthesis of AMP from IMP (Fig. 1b), generating adenylosuccinate, as follows: IMP + aspartate + GTP (Mg^2+^) → adenylosuccinate + GDP + orthophosphate. In the second step, adenylosuccinate lyase cleaves adenylosuccinate to form AMP. AdSS operates at a branch point of the purine salvage pathway and the de novo synthesis of purines (Fig. 1a, b).

Liechti and Goldberg (2012) found that a Δ*purA* mutant (missing the gene encoding for adenylosuccinate synthetase, AdSS) is capable of growth only in medium supplemented with adenine or adenosine, and that its growth is significantly retarded in nutrient-rich medium. The data indicate the essentiality of AdSS for survival of *H. pylori*. In contrast, deletion of the putative nucleoside phosphorylase (PNP) gene, *deoD*, resulted in an inability of *H. pylori* to grow on purine nucleosides or the purine base adenine. These results suggest that inhibiting these two enzymes, PNP and AdSS, may cause an additive, or even a synergistic, effect in *H. pylori* (Pillai et al. 2005).

**Fig. 2:**
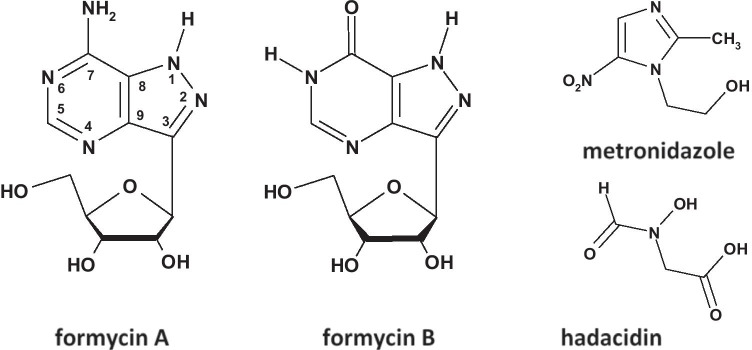
Inhibitors studied in this report, formycins A and B, hadacidin and metronidazole. Formycins A and B are structural analogues of natural PNP substrates, adenosine and inosine, respectively, with the C–C bond linking purine base and the sugar moiety, instead of the N–C glycosidic bond present in adenosine and inosine. Note the difference in base ring numbering of formycins when compared with purine ring numbering. In contrast to natural PNP substrates, adenosine, inosine, and guanosine, formycins may exist in more than one tautomeric form. Here the structure of N(1)-H tautomer is shown (Wierzchowski and Shugar 1982; Bzowska et al. 1992). Hadacidin is a structural analogue of one of the AdSS substrates, aspartate, and is a potent inhibitor of this enzyme (Iancu et al. 2006; Bubić et al. 2018). Metronidazole is an antibiotic efficiently used in therapy against *H. pylori* (e.g., Min Kim et al.; Olmedo et al. 2020), and it is used in this study as a positive control of the cellular uptake

Given the importance of purine production and its direct effect on bacterial growth rates, and taking into account all aspects raised above, targeting enzymes of the salvage pathway seems to be a promising approach for therapy against *H. pylori*. The main objectives of the current project, which we initiated some time ago, are to understand molecular mechanisms of these two key enzymes, PNP and AdSS of the purine salvage pathway of this bacterium, and ascertain whether this knowledge may be useful to eradicate *H. pylori* using newly designed drugs. We have already described catalytic properties of *H. pylori* purine nucleoside phosphorylase, identified potent inhibitors, and obtained the 3D structure of this enzyme complexed with one of its nucleoside inhibitors, formycin A (see Fig. 2) (Narczyk et al. 2018). Simultaneously, we have obtained recombinant *H. pylori* adenylosuccinate synthetase and characterized its biochemical and kinetic properties (Bubić et al. 2018), and recently we have also determined the 3D structure of this enzyme in a complex with its potent inhibitor, hadacidin (Fig. 2) (unpublished, PDB 6ZXQ).

In the present manuscript, we report properties of one more nucleoside PNP inhibitor, formycin B; the effects of all three compounds, formycin A, formycin B, and hadacidin, on *H. pylori* growth as determined by their minimal inhibitory concentrations, MIC; and the possible synergy of PNP and AdSS inhibitors, as evaluated by the fractional inhibitory concentration index (FICI) (Pillai et al, 2005). To understand reasons for discrepancies between observed effects in vivo and in vitro, we have measured the cellular uptake of these inhibitors. To that end we developed, based on Zhou et al. (2015), a universal method suitable a priori for checking bacterial cellular uptake of any possible inhibitor, not only radiolabeled or fluorescently labeled compounds. This method will be extremely useful in further studies of cellular penetration of any inhibitor of *H. pylori* enzymes, not only those belonging to the purine salvage pathway, and the method may also be adapted to study cellular uptake of drugs by any other bacteria.

## Materials and methods

### Materials

Metronidazole (MW 171.15 g/mol), inosine 5′-monophoshate disodium salt hydrate (IMP, MW 392.17 g/mol), guanosine 5′-triphosphate sodium salt hydrate (GTP, MW 523.18 g/mol), L-aspartic acid sodium salt monohydrate (MW 173.10 g/mol), L-arginine-5-13C,4,4,5,5-d_4_ (MW 179.22 g/mol), magnesium chloride, amoxicillin, and Nutrient Mixture F12 Ham medium were obtained from Sigma-Aldrich (Saint Louis, MO, USA). Formycin A (monohydrate, MW 285.27 g/mol) and formycin B (MW 268.23 g/mol) were purchased from Berry & Associates (Dexter, MI, USA). Hadacidin (MW 119.08 g/mol) was purchased from Anji Biosciences (Hyderabad, India). 7-Methylguanosine (m^7^Guo) was synthesized from guanosine according to Jones and Robins (1963) using the method involving methyl iodide. This yields preparation free of sulfate, which as an ion resembling phosphate, a purine nucleoside phosphorylase substrate, could bias the results. Partition coefficients between the lipid and aqueous phases of the neutral, log P, and ionized, log D, forms of the tested compounds, and of metronidazole used as a control in the cellular uptake studies, were calculated using the Molinspiration Cheminformatics website http://www.molinspiration.com.

Recombinant purine nucleoside phosphorylase (PNP) and adenylosuccinate synthetase (AdSS) from *H. pylori* 26695 strain were obtained as described previously (Bubić et al. 2018; Narczyk et al. 2018).

Culture reagents Fetal Bovine Serum, *Helicobacter pylori* Selective Supplement, Brain Heart Infusion Broth (BHI medium) and horse serum (5%), New Zealand Origin, heat inactivated, were from Thermo Fisher Scientific (Waltham, MA, USA). Yeast extract (2.5%) and Christensen’s urea broth were from Torlak (Serbia) and BD IsoVitaleX (1%) from Becton, Dickinson and Company (Franklin Lakes, NJ, USA).

Atmosphere generators were purchased from Biomerieux, France, and from Mart Microbiology B.V., The Netherlands (Anoxomat Mark II). 24-deep-well 2 mL plates, flat-bottomed for suspension cultures used to evaluate *H. pylori* growth under various conditions, were from Nest Scientific Biotechnology (NJ, USA). The *H. pylori* wild-type strain 26695 was obtained from ATCC (Manassas, VA, USA), while the SS1 strain was a mouse-adapted *H*. *pylori* isolated from a patient with peptic ulcer disease (Touati et al. 2000).

### Inhibition constant for formycin B vs. *H. pylori* PNP

The inhibition constant for formycin B vs. 26695 *H. pylori* strain PNP was determined at 25 °C, in 50 mM Hepes/NaOH pH 7.0, 50 mM phosphate, with m^7^Guo as a variable substrate, using the initial velocity method and spectrophotometric assay at λ_obs_ = 260 nm, as described earlier for inhibition constant of formycin A vs. the same enzyme (Narczyk et al. 2018). Kinetic experiments with PNP, and also with AdSS, described in the section “AdSS and PNP inhibition detection of cellular uptake”, were performed on a double-beam UV/VIS spectrophotometer Cary 100, with thermostated Peltier cell holders (Varian: Agilent Technologies, Mulgrave, Vic., Australia). The inhibition constant was determined as the adjustable parameter in the global fitting of the competitive inhibition model to all data obtained, using the GraphPad Prism program (GraphPad Software, San Diego, CA, USA).

### Minimal inhibitory concentration (MIC)

Minimal inhibitory concentration of inhibitors was determined by an in vitro broth microdilution assay (Knezevic et al. 2018). Briefly, double-strength Brain heart infusion broth (BHI) was supplemented with yeast extract (2.5%), horse serum (5%), and IsoVitaleX (1%). In each well, twofold serial dilutions of inhibitors (from 1000 µg/mL to 7.8 μg/mL in a final volume) and an equal volume of inoculated double-strength BHI were added (approx. 1 × 10^6^ CFU/mL in final volume). Amoxicillin was used as a positive control, and distilled water as a negative control. The microtiter plates were incubated at 37 °C under microaerobic conditions in moist atmosphere for 3 days. For easier MIC determination, the method was improved by addition of the equal volumes of double-strength Christensen’s urea broth into wells after incubation, and the plates were additionally incubated 4 h in an aerobic atmosphere at 37 °C. During the incubation, in wells with viable *H. pylori* urease produced by the bacteria converted urea into ammonia and carbon dioxide, changing the pH and color of a phenol red indicator in the medium (from orange to purple). The plates were examined visually for color change and OD_560_ was measured after 4 h in order to determine presence/absence of growth. The MIC is defined as the lowest concentration of a compound that inhibits visible growth, compared with the growth in the controls (in this case defined as presence/absence of color change). All experiments were performed in duplicate and in 3–6 independent tests.

### Inhibitor combinations

In order to determine possible additive or even synergistic effect of inhibitors, a method with calculation of the fractional inhibitory concentration index (FICI) was used (Pillai et al, 2005). A combination of two inhibitors was applied (w/w 1:1) in the final concentration 1 mg/mL and subsequently diluted. The FICI index was calculated as FICI = (MIC_A_/MICa) + (MIC_B_/MICb), where MIC_A_ = concentration of inhibitor a in combination with inhibitor b; MICa = MIC of inhibitor a, applied as a single agent; MIC_B_ = concentration of inhibitor b in combination with inhibitor a; and MICb = MIC of inhibitor b applied as a single agent. The results were interpreted as a synergistic effect if the FICI was ≤ 0.5; as an additive effect if 0.5 < FICI ≤ 1; an indifferent effect if 1 < FICI ≤ 4; and as an antagonistic effect if the FICI was > 4 (Pillai et al. 2005).

### Cellular uptake experiments

Cellular uptake of PNP inhibitors, formycin A (FA) and formycin B (FB), and the control compound, metronidazole (MTZ), was determined using the approach described by Zhou et al. (2015) for *E. coli* and *Pseudomonas aeruginosa*, adapted to conditions suitable for *H. pylori* cell growth. This method measures the change of extracellular drug concentration using LC–MS detection. We also developed additional methods of detection, since LC–MS was not suitable for hadacidin (Hada) (see below).

Overnight cultures of *H. pylori* wild-type 26695 strain were grown by shaking (120 rpm) at 37 °C under microaerophilic conditions (5% O_2_, 10% CO_2_, 85% N_2_) in BHI medium (37 g/L) supplemented with 10% Fetal Bovine Serum and 1% *Helicobacter pylori* Selective Supplement. Cells were collected by centrifugation (5 min at 8000 × *g* at room temperature). The supernatant was decanted and the cell pellets were suspended in F12 medium (with L-glutamine and sodium bicarbonate) and centrifuged again. The supernatant was decanted again and cell pellets were resuspended in such a volume of F12 medium to obtain an OD_600_ of 4.0. F12 medium was selected for experiments because it is a minimal medium of defined composition and is suitable for measurements on MS.

**Fig. 3:**
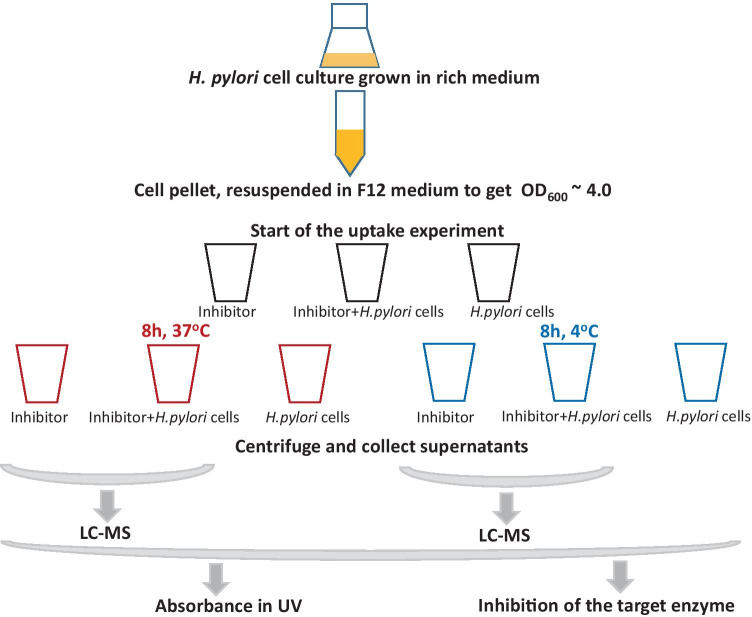
Graphical description of the inhibitor accumulation experiments. The key idea of the method is to measure the change of extracellular inhibitor concentration, after incubation of the compound in the presence of *H. pylori* cells, to estimate the intracellular accumulation of the tested compound. Quantification of the extracellular concentration of inhibitors in the supernatants was done with the aid of one or more detection methods: ultra-performance liquid chromatography–tandem mass spectrometry, UV absorption measurement or determination of the inhibition of the target enzyme for the tested compound, as described in “Materials and methods” (based on Zhou et al. 2015)

The accumulation test was carried out in 24-deep-well 2 mL flat-bottomed plates, all in a final volume of 1 mL. Three types of samples were used, generated by mixing the following components in a 1:1 ratio: (1) concentrated and resuspended cells in F12 medium, (2) a solution of the appropriate concentration of a test compound in F12 medium, (3) F12 medium. Mixing (1) and (2) gave the basic samples (named “Inhibitor + *H. pylori* cells”), while mixing (1) with (3), and (2) with (3), gave two types of the control samples, called “*H. pylori* cells” and “Inhibitor,” respectively (Fig. 3).

The final concentration of the metronidazole, used as a positive control of the uptake process, was 0.5 µM (80 ng/mL); this is a 100-times lower concentration than the MIC value — minimal inhibitory concentration (according to EUCAST 2020). The final concentrations of the tested compounds were 350 µM and 35 µM for formycin A and formycin B, and 35 μM for hadacidin. One set of three replicate samples was incubated at 37 °C, and the other set was incubated at 4 °C. Samples were taken at the start of the accumulation test, to check the concentration of the inhibitor at the start (named “Inhibitor_start,” “*H. pylori* + inhibitor_start”), then after 4 and 8 h of incubation at both temperatures. The obtained samples were centrifuged at 8000 × *g* for 5 min at 4 °C. The supernatant was collected for the LC–MS analysis (used for MTZ, FA, and FB uptake), UV absorption measurements (used for FA and FB uptake), and AdSS and PNP inhibition analysis (used for Hada and FB uptake).

The samples incubated in the cold condition (4 °C) were included to account for nonspecific binding of tested compounds to the surface of the bacterial cell wall.

The samples of the inhibitor in the F12 medium, without cells (“Inhibitor”) at both temperatures, were included to check solvent evaporation, compound degradation, and nonspecific binding of the compound to the plates.

The samples of the cells in the F12 medium, without inhibitor (“*H. pylori* cells”) at both temperatures, were included to check cellular uptake or release of some compounds from or to the medium that may interfere with UV absorption and enzyme inhibition measurements.

The differences observed between the samples of the inhibitor, incubated with and without *H. pylori* cells, at cold 4 °C and warm 37 °C conditions, were used to estimate the intracellular accumulation of the tested compound, as described by Zhou et al. (2015):

1 $$\begin{aligned}&C_{accumulated\ intracellular}\\&=(C_{inhibitor8h37^o C}-C_{(Hp+inhibitor)8h37^o C})\\&-(C_{inhibitor8h4^o C}-C_{(Hp+inhibitor)8h4^o C}) \end{aligned}$$

C corresponds to inhibitor concentration, and subscripts “inhibitor” and “Hp + inhibitor” refer to the control without cells and samples of the inhibitor incubated in the presence of *H. pylori* cells, respectively (see above), while subscript 37 °C and 4 °C refer to two temperature conditions used.

This simple form of the equation was used for LC–MS detection, which is most specific of the three methods we used. For the UV absorption and inhibition of the target enzyme detection of the cellular uptake of tested inhibitors (see below), samples with *H. pylori* cells in F12 medium incubated for 4 and 8 h at both temperatures were also included (“*H. pylori* cells,” see above). These samples were necessary to make correction of the results for the effects caused by uptake of some nutrients from the medium by *H. pylori* cells, as well as by release of some metabolic products, which may exhibit absorption in UV–VIS range and may inhibit target enzyme. In these cases, Eq. (1) was extended to account for these effects and this is described in detail in next sections (see Eq. (5) below).

### LC–MS detection of cellular uptake

Formycin A, formycin B, and metronidazole were quantified through the ultra-performance liquid chromatography–tandem mass spectrometry. Samples were analyzed on Waters Xevo TQ-S with a standard ESI ion source coupled to Waters Acquity I-class UPLC.

Calibration samples were prepared by spiking blank growth medium F12 by the tested inhibitor, formycin A, formycin B, or metronidazole solutions. Internal standards (IS) were used to improve the accuracy of the method. To each pipetted sample from a particular experiment, the same amount of IS was added. As a detector response, we considered the ratio of the analyte peak area (variable) to the internal standard peak area (constant). This allows compensation for signal changes due to a possible instability of the ion source. If the ionization efficiency changes, both compounds are affected, but the surface peak ratio is maintained. As internal standards, compounds that do not occur naturally in *H. pylori* were used, namely formycin A, when analyzing formycin B, and vice versa, and deuterated arginine when metronidazole uptake was determined. Samples and calibrators were precipitated using IS solution and injected on the instrument.

Calibration curves were acquired by plotting the concentration of the standards against the ratio of the analyte peak area to the IS peak area. The calibration curve for FA in low concentration (35 μM) consisted of 7 samples in the range from 0.23 to 37.5 μM (*R*^2^ = 0.997); for FA in high concentration (350 μM), the curve consisted of 6 samples in the range from 3.7 to 374.7 μM (*R*^2^ = 0.995); for FB in low concentration (35 μM), the curve consisted of 7 samples in the range from 0.291 to 46.5 μM (*R*^2^ = 0.999); for FB in high concentration (350 μM), the curve consisted of 7 samples in the range from 2.91 to 465.0 μM (*R*^2^ = 0.999); and for MTZ (0.5 μM), the calibration curve was 8 samples in the range from 0.584 to 584.2 nM (*R*^2^ = 0.995).

**Fig. 4:**
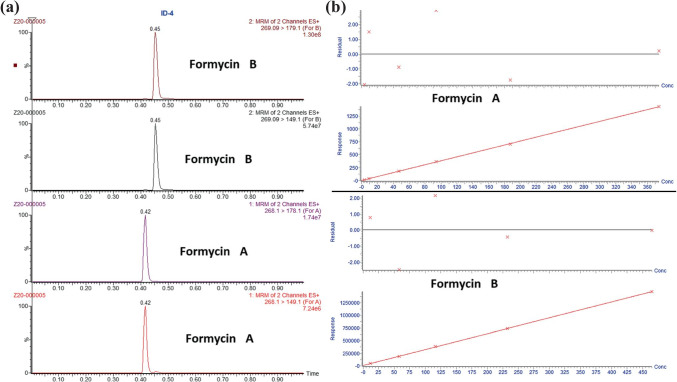
(a) Example of chromatograms of formycin A (two lower chromatograms) and formycin B (two upper chromatograms) obtained in the quantification of both compounds in the cellular uptake experiments using ultra-performance liquid chromatography–tandem mass spectrometry, showing their retention time under conditions described in “Materials and methods”. (b) Two upper panels: calibration curve and residuals plot formycin A. Two lower panels: calibration curve and residuals plot formycin B

Chromatography was run on Waters Acquity BEH C18, 1.7 μm, 2.1 mm × 50 mm analytical column thermostated at 70 °C. The separations were done using 2.5 min gradient method. Phase A was 0.1% formic acid in MilliQ water; phase B was 0.1% formic acid in acetonitrile. For formycins the gradient started at 5% B and increased linearly to 50% B in 1.6 min; at 1.8 min it returned to the starting condition for column equilibration. For metronidazole the gradient started at 5% B and increased linearly to 50% B in 1.3 min; at 2.0 min it returned to the starting condition for column equilibration. The flow rate was 600 μL/min in both methods. The retention time of FA was 0.42 min, FB was 0.45 min (see Fig. 4a, b), and MTZ 0.88 min.

MS was working in positive polarity mode, the capillary voltage was 3 kV, desolvation temperature 550 °C, and desolvation gas flow 900 L/h. FA was analyzed using two transmissions 268.19 > 149.1 (Collision energy 32) and 268.19 > 178.1 (Collision energy, CE 20); FB 269.09 > 149.1 (CE 25) and 269.09 > 179.1 (CE 17); MTZ 172.07 > 82.00 (CE 20) and 172.07 > 128.00 (CE 15); deuterated arginine 180.12 > 75.07 (CE 55).

### AdSS and PNP inhibition detection of cellular uptake

Cellular uptake of hadacidin was tested in a similar experiment as FA, FB, and MTZ, but the detection method was different. Inhibition of AdSS was used, since the hadacidin molecule is too small and too volatile for the LC–MS approach.

AdSS catalyzes utilizing three substrates: inosine 5′-monophoshate (IMP), guanosine 5’-triphosphate (GTP), and aspartate (Asp). The enzyme specific activity was determined for the purified protein, at pH 7.7, in 20 mM Hepes/NaOH buffer at 25 °C. The reaction mixture contained 0.15 mM IMP, 5 mM Asp, 0.06 mM GTP, and 1 mM MgCl_2_ in the above-mentioned buffer. The reaction progress was monitored spectrophotometrically by adding AdSS (approximately 20 nM) to 1 mL of the reaction mixture (thermostated for 5 min) and by measuring the change in absorption at 280 nm for 3 min. The extinction coefficient used to calculate the amount of the products formed is ε_280_ = 11,700 M^−1^ cm^−1^ (formation of adenylosuccinate) (Rudolph and Fromm 1969). One unit (U) of AdSS enzymatic activity is defined as µmol of adenylosuccinate formed per min at 25 °C. Specific activity is expressed as units per mg of protein (U/mg).

For detection of cellular uptake of hadacidin, which is the AdSS inhibitor competing with Asp (Bubić et al. 2018), the Asp concentration in the assay was lowered to 0.1 mM. Twenty microliters of each supernatant was added to the reaction mixture. The control reactions with 20 μL of F12 medium (“medium”), and with 20 μL of *H. pylori* cells incubated for 8 h (“Hp cells”), were done. The activity observed in the latter case was treated as the reference, observed in the absence of the inhibitor. Values obtained for samples with the inhibitor in F12 medium at the start of the accumulation test (“inhibitor_start”) were treated as containing the starting inhibitor concentration (in our case 35 µM of hadacidin). Average activity observed for these samples was used to calculate the hadacidin concentration in all other samples, in particular, samples incubated for 4 and 8 h at 37 °C and at 4 °C containing either hadacidin in F12 medium, or hadacidin in F12 medium and *H. pylori* cells (“inhibitor” and “Hp + inhibitor”, respectively).

Since hadacidin is an inhibitor of AdSS that competes with Asp, and since the Michaelis–Menten model properly describes the reaction (Bubić et al. 2018), the following equations show the dependence of initial velocity of the reaction on initial substrate concentration, *S*_*o*_, observed in the supernatant containing only F12 medium, *v* (Eq. (2)), and containing the inhibitor, in the concentration *C*, in F12 medium, *v*_*i*_ (Eq. (3)):

2 $$v\left({S}_{o}\right)=\frac{{{V}_{\mathrm{max}}S}_{o}}{{{S}_{o}+K}_{\mathrm{m}}}$$

3 $$v\left({S}_{o}\right)=\frac{{{\mathit{V}}_{\mathrm{max}}S}_{o}}{{{S}_{o}+(1+ \frac{C}{{K}_{\mathrm{i}}})K}_{\mathrm{m}}}$$

where *K*_m_ and *V*_max_ are the Michaelis constant and maximal velocity, respectively, and *K*_i_ is the inhibition constant of the tested inhibitor, in our case hadacidin. At the start of the cellular uptake reaction Eq. (3) applies, and the initial velocity, ${v}_{inhibitor\_start}$, and the inhibitor concentration, ${C}_{inhibitor\_start}$, are known. These values, together with velocity measured for any tested supernatant, ${C}_{inhibitor\_start}$, and velocity measured when only F12 medium is present, *v*, are sufficient to determine inhibitor concentration, ${C}_{tested}$, present in a tested supernatant, using Eq. (4), which is obtained by the rearrangement of Eq. (2) and Eq. (3):

4 $${C}_{tested}={C}_{inhibitor\_start}\left(\frac{1}{{v}_{tested}}-\frac{1}{v}\right)/\left(\frac{1}{{v}_{inhibitor\_start}}-\frac{1}{v}\right)$$

In fact, it is not necessary to know the explicit values of *K*_m_, ${V}_{max}$, and *K*_i_ to use this method, since none of these parameters is present in Eq. (4). It is only important that velocity of the reaction for the sample obtained at the start of the experiment (“inhibitor_start”) and all other samples are assayed under the same conditions.

The formycin B uptake was studied in the same way, but instead of AdSS, PNP was used, since it is the target enzyme for this inhibitor. Other conditions were the following: 25 °C, 50 mM Hepes/NaOH pH 7.0 buffer, 13 nM PNP, 50 mM phosphate and 60 μM m^7^Guo as substrates, λ_obs_ = 260 nm, extinction coefficient to calculate product formation ε_260_ = 4600 M^−1^ cm^−1^ (Kulikowska et al. 1986). One unit (U) of PNP enzymatic activity is defined as µmol of product, in our case 7-methylguanine, formed per min at 25 °C. Specific activity is expressed as units per mg of protein (U/mg).

### UV absorption detection of cellular uptake

**Fig. 5:**
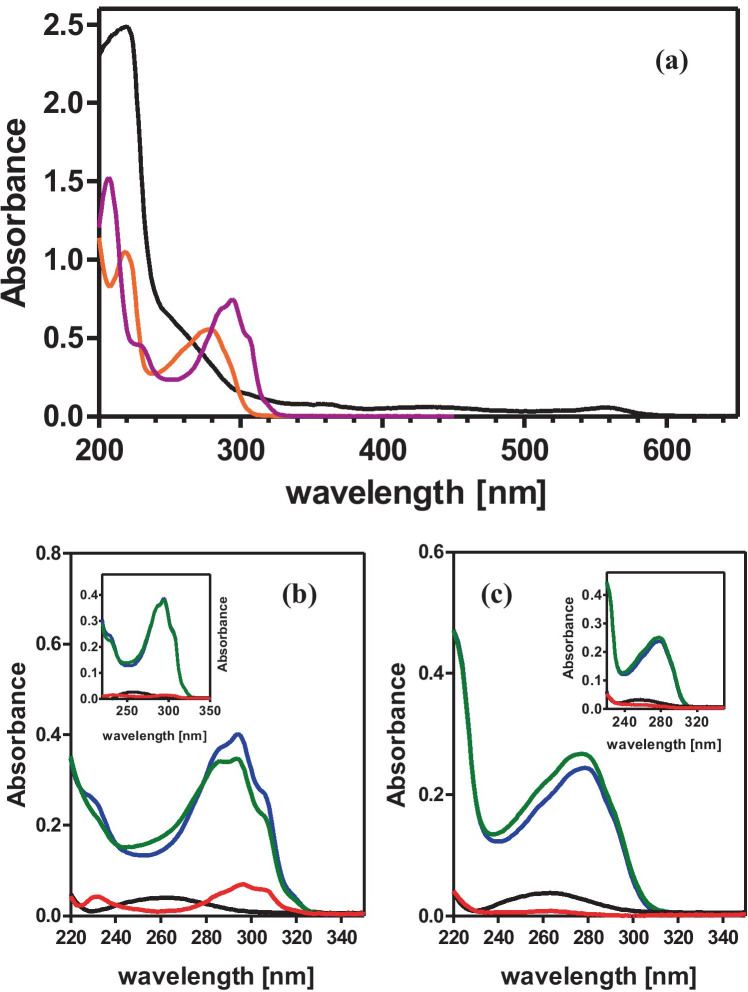
(a) UV spectra of F12 medium ten-fold diluted with water (black), and 70 µM water solution of formycin A (magenta), and formycin B (orange). (b) Cellular uptake of formycin A, and (c) formycin B, both at concentrations of 350 µM, by *H. pylori* 26695 strain after 8 h incubation, quantified by UV absorption spectra of ten-fold water diluted supernatants obtained from the cellular uptake experiment (see text for details of the method). F12 medium contained the following supplements: inhibitor (blue), inhibitor and *H. pylori* 26695 strain (green), *H. pylori* 26695 strain cells (black), and difference spectra (red), obtained by subtraction of green and black spectra from the blue (blue spectrum-green spectrum-black spectrum), which shows the inhibitor uptake. Main figure and the respective insert show data obtained for samples incubated at 37 °C and at 4 °C, respectively

For cellular uptake of formycin A and formycin B, UV absorption was additionally used as a detection method. This was possible since UV absorption of formycins is shifted to a longer wavelength compared with the main UV absorption bands of the F12 medium, exhibiting maximum of around 220 nm (Fig. 5a). Nevertheless, there is some overlap between these spectra, and because of that, selective observation of formycins absorption is not possible. Moreover, during the cellular uptake experiment, *H. pylori* cells may take some UV absorbing nutrients from the medium, as well as release some metabolic products that also absorb in the UV region. Therefore, in the cellular uptake experiment, correction for absorption caused by these processes was introduced by including samples containing just *H. pylori* cells in F12 medium, incubated for 4 and 8 h at both temperatures, and subtracting absorption spectra of such supernatants. Therefore, in this case Eq. (1) proposed by Zhou et al. (2015) modifies to:

5 $$\begin{aligned}&C_{accumulated\ intracellular}\\&=\left[(A_{inhibitor8h37^o C}- A_{(Hp+inhibitor)8h37^o C}- A_{Hp8h37^o C})\right.\\&-\left.(A_{inhibitor8h4^o C} - A_{(Hp+inhibitor)8h4^o C} - A_{Hp8h4^o C})\right]/{\epsilon} \end{aligned}$$

where C corresponds to inhibitor concentration, A, to the absorption of the supernatant in the chosen observation wavelength, and ε, to the inhibitor extinction coefficient at this wavelength.

Subscripts “inhibitor,” “Hp + inhibitor,” and “Hp” refer to blank control without cells, samples of the inhibitor incubated in the presence of *H. pylori* cells, and control with *H. pylori* cells in F12 medium, while subscripts 37 °C and 4 °C refer to two temperature conditions used.

Samples in this case were the same as those evaluated by the LC–MS detection and by analysis of the inhibition of the PNP activity. The UV spectra of the supernatants, from experiments with 350 μM and at 35 μM inhibitor concentration, diluted with water 10 times and 2 times, respectively (with the absorbance of F12 medium, 10 times or 2 times diluted with water, as a reference), were measured. The absorbance at the most suitable wavelengths was used to calculate the formycin concentration. The following extinction coefficients were used: for FA ɛ_294_ = 10300 M^−1^ cm^−1^ and ɛ_305_ = 7100 M^−1^ cm^−1^, for FB ɛ_279_ = 7300 M^−1^ cm^−1^ and ɛ_294_ = 4150 M^−1^ cm^−1^. Coefficients for maxima, 294 nm for FA and 279 nm for FB, are from Bzowska et al. (1992), while those for 305 nm for FA and 294 for FB were calculated on the basis of the UV spectrum of FA and FB in 10% F12 medium (not shown).

### Statistical analysis

Data from the cellular uptake experiments were expressed as means ± SEM. A Student’s *t*-test was used to determine if there was a significant difference between the means of two groups, it means in supernatants incubated with and without *H. pylori* cells, and in supernatants with *H. pylori* incubated at 4 °C and in 37 °C. The GraphPad Prism program was employed to perform these analyses.

## Results

### Inhibition of *H. pylori* PNP by formycin B

**Fig. 6:**
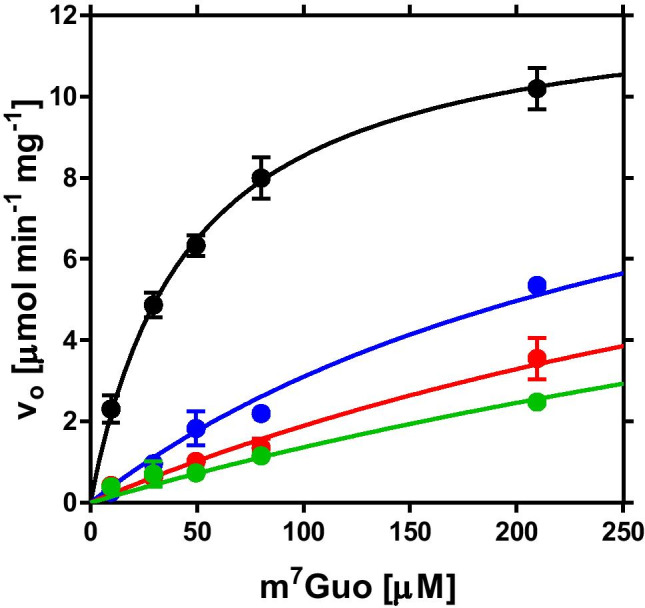
Inhibition of *H. pylori* PNP by formycin B, at 25 °C, in 50 mM Hepes/NaOH pH 7.0, 50 mM phosphate, with m^7^Guo as a variable substrate. Initial velocity, *v*_o_, vs. substrate concentration is presented, with no inhibitor added (black circles) and with 5.33 µM (blue), 10.66 µM (red) and 15.99 µM (green) formycin B. Error bars show standard errors from two experiments. Lines show the global fit of the competitive inhibition model with *K*_i_ = 0.96 ± 0.08 µM, *K*_m_ = 46.3 ± 4.4 µM, and *V*_max_ = 12.5 ± 0.5 µmol min^−1^ mg^−1^

We have previously shown that formycin A, the analogue of adenosine with C–C bond between the base and the pentose (Fig. 2), is an efficient inhibitor of purine nucleoside phosphorylase (PNP) from the *H. pylori* 26695 strain. The inhibition constant, *K*_i_ = 14.0 ± 1.7 μM (Narczyk et al. 2018), is about threefold higher than that reported for *E. coli* PNP, *K*_i_ = 5.3 ± 0.4 μM (Bzowska et al. 1992). Thus, we decided to check another *E. coli* PNP inhibitor, formycin B, the inosine analogue with C–C bond between the base and the pentose (Fig. 2), vs. PNP from *H. pylori* 26695 strain (Fig. 6). We found that the inhibition constant in this case is *K*_i_ = 0.96 ± 0.08 µM. Hence, formycin B turned out to be a several-fold more effective inhibitor of *H. pylori* PNP than formycin A.

### *H. pylori* sensitivity to hadacidin, formycin A, and formycin B

Since formycin A (FA), formycin B (FB), and hadacidin (Hada) are good in vitro inhibitors of *H. pylori* 26695 strain PNP and AdSS, respectively, we decided to check their impact on *H. pylori* 26695 strain growth. Although in vitro inhibition constants observed are *K*_i_ = 0.19 ± 0.02 μM for hadacidin vs. AdSS (Bubić et al. 2018), and 14.0 ± 1.7 μM and 0.96 ± 0.08 µM for FA (Narczyk et al. 2018) and FB vs. PNP, these compounds do not significantly affect growth of *H. pylori* 26695 and SS1 strains in vivo in concentrations up to 1000 μg/mL, which corresponds to several mM (Table 1). Thus, the MIC is at least orders of magnitude higher than the inhibition constants vs. target enzyme.

**Table 1:** MIC values of formycin A (FA), formycin B (FB), and hadacidin (Hada) against *H. pylori* strains*.* The results are presented as the geometrical mean of repetitions

Inhibitors	*H. pylori* ATCC 26695 strain				*H. pylori* SS1 strain		
	*K*_i_ (µM)	MIC (µg mL^−1^)	MIC (mM)	FICI	MIC (µg mL^−1^)	MIC (mM)	FICI
FA	14.0 ± 1.7	≥ 870	≥ 3.1	-	> 1000	> 3.5	-
FB	0.96 ± 0.08	> 1000	> 3.7	-	≥ 397	≥ 1.5	-
Hada	0.19 ± 0.02	> 1000	> 8.4	-	> 1000	> 8.4	-
FA/FB 1:1	-	500/500	1.8/1.9	1.1	177/177	0.6/0.7	0.6
FA/Hada 1:1	-	> 500/500	> 1.8/4.2	> 1.1	> 500/500	> 1.8/4.2	> 1.0
FB/Hada 1:1	-	> 500/500	> 1.9/4.2	> 1.0	> 500/500	> 1.9/4.2	> 1.8

The impact of inhibitor combinations was also examined. This was achieved by determining the fractional inhibitory concentration index (FICI), according to Pillai et al. (2005), as described in “Materials and methods”. The data revealed neither synergistic nor additive effect in combination 1:1 (w/w), with an exception of FA and FB combination against the strain *H. pylori* SS1. In this case, the effect was additive, as indicated by FICI = 0.62 (Table 1).

The possible reason for such a discrepancy between in vitro and in vivo effects could be the poor penetration of inhibitors into bacteria cells. Therefore, we decided to check cellular uptake of formycin A, formycin B, and hadacidin by the *H. pylori* 26695 strain.

### Versatile method for determining cellular uptake of inhibitors by *H. pylori*

In order to check how inhibitors examined in this study penetrate *H. pylori* bacterial cells, a universal method for determining cellular uptake of such compounds by *H. pylori* was developed. The idea of Zhou et al. (2015), proposing a unique assay to measure intracellular drug penetration in gram-negative bacteria, was adopted for *H. pylori* (Fig. 3)*.* Moreover, two alternative detection methods, in addition to the liquid chromatography (LC)–mass spectrometry (MS) approach exploited by Zhou et al. (2015), were shown to be applicable in monitoring decrease of extracellular inhibitor concentration as a result of inhibitor accumulation in the cells, which is the key idea of this method (Fig. 3).

*H. pylori* cells were grown in a rich medium, and centrifuged and resuspended in F12 minimal medium to obtain an OD_600_ of 4.0. Two samples of bacteria in the medium containing the tested inhibitor, at a concentration lower than its MIC value, were incubated, one in warm (37 °C) and the other in cold (4 °C) conditions. These were compared to the blanks containing the same concentration of the inhibitor in F12 medium (without cells) at these two temperatures. The controls without cells at both temperature conditions documented possible solvent evaporation, compound degradation, and/or nonspecific binding of the compound to the plates. The control with *H. pylori* cells incubated in the cold condition (4 °C) was also done, as it additionally accounts for a nonspecific binding of tested compounds to the surface of the bacterial cell wall. Finally, the controls with *H. pylori* cells in F12 medium (without the inhibitor) incubated in warm and in cold conditions indicate changes in the medium caused by the presence of *H. pylori* cells, which may utilize some medium components as well as release some products of their metabolism to the medium.

The starting concentrations of tested compounds were as follows: 350 μM and 35 μM for formycin A and formycin B, 35 μM for hadacidin, and 0.5 μM for the positive control compound, metronidazole. Samples were taken at the start of the accumulation test, and then after 4 and 8 h of incubation at both temperatures. The samples were centrifuged, and the supernatants were collected for direct measurements of extracellular inhibitor concentration.

Three detection methods were used to measure the extracellular concentration of tested inhibitors: UV absorption, proposed more than 40 years ago by Nikaido (1976); LC–MS, used by Zhou et al. (2015); and the inhibition of a target enzyme, which was our original idea, developed and tested in this study. Results presented below were obtained for the cells incubated for 8 h. In principle, similar results were obtained after 4 h incubation; however, since the effects were smaller and the errors larger, 8 h of incubation was better for measuring cellular uptake.

The differences observed for the samples of the inhibitor incubated with and without *H. pylori* cells, at cold, 4 °C, and warm, 37 °C, conditions, were employed to estimate the cellular uptake of the tested compound using Eq. (1). If correction for metabolites released by *H. pylori* was necessary, Eq. (5) was used.

### Extracellular inhibitor concentration quantified by LC–MS

Ultra-performance liquid chromatography–tandem mass spectrometry (LC–MS) is a very sensitive and accurate method to measure the extracellular inhibitor concentration. Calibration was done using the tested inhibitor, with the curves consisting of 6–8 points spanning the concentration range from about 10% higher than the maximal concentration used in the cellular uptake experiment (to capture the point expected when no penetration is taking place) to about 100-fold lower (expected in supernatant if practically all inhibitor penetrates the cells). Calibration samples were prepared by spiking blank growth medium F12 with the tested inhibitors, formycin A, formycin B, or metronidazole solutions. As internal standards, compounds that do not occur naturally in *H. pylori* were used, namely formycin A when analyzing formycin B, and vice versa, and deuterated arginine when metronidazole uptake was determined. Samples and calibrators were precipitated using internal standard solution (IS) and injected on the instrument. Calibration curves were acquired by plotting the concentration of the standards against the ratio of the analyte peak area to the IS peak area. Correlation coefficients *R*^2^ for each standard curve were at least 0.99.

**Fig. 7:**
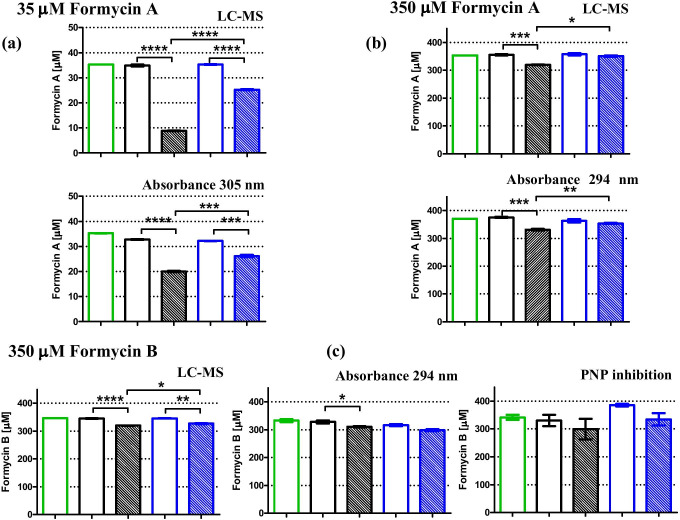
Cellular uptake of formycin A (a) (b) and formycin B (c) by the *H. pylori* 26695 strain after 8 h of incubation, quantified by various methods of detection, LC–MS, UV absorbance or inhibition by a target enzyme, purine nucleoside phosphorylase. In the case of the absorbance method, shifting observation to the longer wavelength allowed monitoring experiments with lower inhibitor concentrations (here shown for 35 µM FA). Green bars show inhibitor concentration observed at the start of the incubation. Black and blue bars show data (extracellular inhibitor concentration) obtained for incubation at 37 °C and 4 °C, respectively. Open bars indicate inhibitor incubated in the F12 medium, while filled bars indicate inhibitor incubated in F12 medium containing *H. pylori* cells. Results are mean ± SEM, *n* = 3, ****p < 0.0001; ***p < 0.001; **p < 0.01, *p < 0.05

**Fig. 8:**
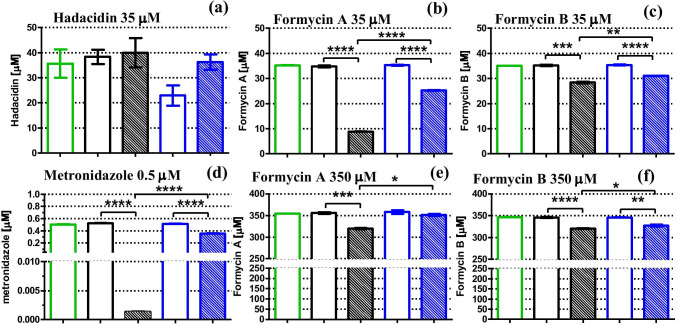
Cellular uptake of formycins A and B (both at 350 µM (e) (f) and 35 µM (b) (c)), hadacidin (35 µM (a)) and metronidazole (0.5 µM (d)) by *H. pylori* 26695 strain after 8 h incubation, quantified by LC–MS detection in all cases, except for hadacidin, which was quantified by inhibition by a target enzyme, AdSS. Black and blue bars show data (extracellular inhibitor concentration) obtained for incubation at 37 °C and 4 °C, respectively. Green bars show inhibitor concentration observed at the start of the incubation. Open bars indicate inhibitor incubated in the F12 medium, while filled bars indicate inhibitor incubated in F12 medium containing *H. pylori* cells. Results are mean ± SEM, *n* = 3, ****p < 0.0001; ***p < 0.001; **p < 0.01, *p < 0.05. The intracellular accumulation of each drug was determined from these data using Eq. (1) or Eq. (5) as described in the text, and the results are shown in Table 2

This detection method was used for formycins, at 35 µM and 350 µM concentrations, and for metronidazole at 0.5 μM concentration, and results are depicted in Figs. 7 and 8, and in Table 2. In all cases, errors of the measured concentration are very low and the uptake calculated from Eq. (1) has errors in the range 3–30% (see Table 2).

**Table 2:** Quantification of the cellular uptake of studied inhibitors by *H. pylori* 26695 strain cells. The extracellular inhibitor concentration was determined by three detection methods: LC–MS, UV absorption spectra of the tested compounds, and inhibition of the target enzyme, as described in the text. The *t*-test was used to check for a significant difference in extracellular inhibitor concentration between supernatants incubated with and without *H. pylori* cells. The intracellular accumulation of each drug was determined using Eq. (1) or Eq. (5) as described in the text. Results of the *t*-test are marked as follows: ****p < 0.0001; ***p < 0.001; **p < 0.01, *p < 0.05; ns *p* > 0.05, not significant difference

Inhibitor	Starting inhibitor concentration [µM]	Method	Observation wavelength [nm]	Intracellular accumulation [µM]	*K*_i_ [µM]	*t*-test
Metronidazole	0.5	LC–MS	-	0.37 ± 0.01		****
Hadacidin	35	AdSS inhibition	-	4.9 ± 4.1	0.19 ± 0.02 vs*.* AdSS^a^	ns
Formycin A	350	UV absorption^c^	305	22.2 ± 7.6	14.0 ± 1.7 vs*.* PNP^b^	*
	350	UV absorption	294	35.2 ± 7.1		**
	350	LC–MS	-	29.5 ± 5.3		*
	35	UV absorption	305	6.7 ± 0.6		***
	35	LC–MS	-	15.9 ± 0.5		****
Formycin B	350	LC–MS	-	7.1 ± 2.5	0.96 ± 0.08 vs*.* PNP	*
	350	UV absorption	279	3.0 ± 9.2		ns
	350	UV absorption	294	1.0 ± 7.4		ns
	350	PNP inhibition	-	20.2 ± 34.0		ns
	35	LC–MS	-	2.5 ± 0.5		**

^a^From Bubić et al. (2018)

^b^From Narczyk et al. (2018)

^c^From Eq. (1), no correction for absorption of metabolites excreted to the medium by *H. pylori* was done

Despite many advantages, LC–MS is not a universal method, and in our research was not appropriate for hadacidin, as this molecule is too small and too volatile for LC–MS. Therefore, we developed a new detection method, described below.

### Extracellular inhibitor concentration quantified by UV absorption

UV absorption in the middle- or in the near-UV region may be used as a detection method only for cellular uptake of compounds that contain UV chromophores absorbing in these spectral regions. Moreover, their absorption band must be shifted to a longer wavelength when compared with the main UV absorption band of the medium in which the cellular uptake experiment is carried out. This method also requires higher dosing concentrations, due to the inherent insensitivity of UV absorption. Even in optimal conditions, the high molar extinction coefficient of the tested compound means that measurement of the UV absorbance will not be able to accurately determine concentrations lower than the μM range. In our case, the concentration of metronidazole was below the sensitivity of this detection method, and hadacidin did not show absorption in the appropriate UV region. In contrast, both formycins, in principle, fulfilled all the required conditions (Fig. 5a), so we used this detection method for each.

UV spectra of the two-fold and ten-fold diluted supernatants, from the cellular uptake experiments with 35 μM and 350 μM formycin concentrations, respectively, were measured, and absorbance at 294 nm (maximum) and 305 nm in the case of formycin A, and 279 nm (maximum) and 294 nm in the case of formycin B, was used to calculate the extracellular concentration of the inhibitor and its potential reduction due to uptake by *H. pylori* cells. One more wavelength, in addition to the maximum, was employed in order to lower the influence of UV absorption band of the F12 medium, partially overlapping the formycin bands (Fig. 5a).

It was noticed that during the 8 h of incubation *H. pylori* cells release some metabolic products to the medium, and these compounds exhibit absorption in the UV region (with a maximum around 260 nm, hence most probably nucleic acid components) as shown in Fig. 5(b, c) (black spectra). Therefore, correction for this absorption was necessary if the observation wavelength was lower than 300 nm, and it was done by using Eq. (5), not Eq. (1), for calculation of the external inhibitor concentration.

Cellular uptake determined by this detection method is presented in Fig. 7(a, b, c) and in Table 2. The formycin A spectrum overlaps less with the main UV absorption band of the F12 medium as compared with the spectrum of formycin B (Fig. 5a). Therefore, good results were obtained for formycin A, at both concentrations studied and for both wavelengths used. In contrast, for formycin B reliable results were obtained only for the higher concentration, 350 μM. The errors of the UV absorption detection method are usually higher than those of the LC–MS detection. The lowest obtained error was about 10% (for 35 μM of formycin A uptake), while only 3% for the latter method (for 35 μM of formycin A and for 0.5 μM of metronidazole uptake, see Table 2). However, when we compared the results of the same uptake experiments, especially when the concentration of the investigated compound in the cells was in the tens of micromoles, e.g., experiments with 350 μM of formycin A, errors of both detection methods were comparable.

### Extracellular inhibitor concentration quantified by inhibition of the target enzyme

Hadacidin is too small and too volatile a molecule for the LC–MS approach, and its UV absorption band overlaps with that of the cell culture media; hence, neither of the methods described above could be used. Therefore, we were forced to develop another method to measure its concentration in supernatants in cellular uptake experiments. Since hadacidin is an inhibitor of AdSS, the concentration of hadacidin present in supernatant samples can be quantified by measuring the ability of the supernatant to inhibit activity of this enzyme. Since hadacidin competes with aspartic acid, its concentration in the enzyme activity assay was therefore lowered to 0.1 mM, slightly below the Michaelis constant for this AdSS substrate (Bubić et al. 2018). This makes the activity test very sensitive to even slight changes in the concentration of hadacidin in the tested supernatants.

The reaction progress was monitored spectrophotometrically by measuring the change in absorption at 280 nm. First, it was documented that the components of the F12 medium do not significantly affect the AdSS activity. Addition up to 5% of the F12 medium (50 µL in 1 mL of the reaction mixture) still allowed accurate measurement of the reaction progress and calculation of the enzyme specific activity. If identical AdSS activities were obtained for supernatant taken from samples incubated at 37 °C with *H. pylori* cells, it would mean the total uptake of the inhibitor. At the other extreme, the appropriately lower enzyme activity obtained for the supernatant taken at the start of the uptake experiment, before the cellular uptake of the inhibitor could start, corresponds to the starting inhibitor concentration. All activity values in between show some uptake, and their particular values were determined using Eq. [4]. No additional corrections for the inhibition by *H. pylori* metabolites excreted to the medium were necessary, since it was found that supernatant from samples with *H. pylori* 26695 after 8 h incubation, tested in the reaction medium up to a 5% concentration, did not inhibit the AdSS-catalyzed reaction.

Results obtained for the uptake of 35 µM of hadacidin, quantified by this detection method, are shown in Fig. 8(a), and in Table 2.

In principle, the approach presented here is suitable for every possible pair of an inhibitor and its target enzyme. Hence, although formycin uptake was already quantified by the LC–MS and UV absorption, we decided to check if inhibition of PNP could be used as a detection method as well.

Formycins compete with nucleosides, specifically with 7-methylguanosine in our assay; therefore, we lowered its concentration in the reaction mixture to 0.05 mM, slightly below the Michaelis constant for this PNP substrate (Narczyk et al. 2018). The reaction progress was monitored spectrophotometrically by measuring the change in absorption at 260 nm. Though the presence of the cell culture media F12 up to 5% in the reaction mixture does not affect PNP activity, *H. pylori* metabolites expelled to the medium do inhibit the PNP catalyzed reaction. Even a 2% concentration of supernatant from an *H. pylori* 26695 strain after 8 h incubation inhibited enzyme activity in the reaction medium 22 ± 4%. Therefore, only strong inhibitors, which inhibit the PNP catalyzed reaction even at a low concentration (when a small volume of the supernatant is added to the reaction mixture), may be quantified for PNP. Formycin B fulfils this condition, as its *K*_i_ is 0.96 ± 0.08 μM. When studied in a 350 μM starting concentration, the respective supernatant present at 0.5% in the reaction medium yielded much stronger inhibition (activity was lowered by more than 50%) than the metabolites excreted by *H. pylori*. The resulting intracellular formycin B concentration determined by this detection method, albeit burdened with greater error, was consistent with results derived with the UV absorption and LC–MS method shown on Fig. 7(c), and in Table 2.

### Cellular uptake of formycin A, formycin B, and hadacidin by the *H. pylori* 26695 strain

Cellular uptakes of formycin A, formycin B, hadacidin, and metronidazole by *H. pylori* were quantified using one or more detection methods, and calculated using Eq. (1) or (5) as described above. Metronidazole, the well-known drug used against *H. pylori*, was employed as a positive control of the uptake, and its concentration was 0.5 μM (80 ng/mL), 100 times lower than its minimal inhibitory concentration (MIC) (according to EUCAST 2020). Since formycins and hadacidin, even in mM concentrations, show only moderate, if any, effect on *H. pylori* growth, their cellular uptake was checked at a concentration of 35 μM, which is about 100 times lower that their estimated MIC values (Table 1), and in the case of formycins also in a ten-fold higher concentration, 350 μM. Results are shown on Fig. 8(a, b, c, d, e, f) and in Table 2.

Metronidazole, in the conditions tested, was almost completely taken inside the bacterial cells, as its intracellular concentration was found to be 0.37 ± 0.01 μM (Fig. 8d, difference observed for the samples with *H. pylori* cells, at cold, 4 °C, and warm, 37 °C, conditions), while the starting concentration was 0.5 μM. This was not the case for formycin A, formycin B, and hadacidin. At 35 μM hadacidin does not enter *H. pylori* cells (Fig. 8a). For both formycins, minimal uptake was observed, as intracellular amounts determined were 2.5 ± 0.5 μM for formycin B, and 15.9 ± 0.5 μM for formycin A (Fig. 8b, c). Moreover, the uptake was not significantly larger when ten-fold more inhibitor was present. When both inhibitors were at 350 μM concentration, the uptake was up to 7.1 ± 2.5 μM of formycin B and up to 29.5 ± 5.3 μM of formycin A (Fig. 8e, f, and Table 2).

## Discussion

So far, loss of the de novo purine nucleotide biosynthesis pathway has been considered a relatively rare evolutionary event. However, due to the increasing number of experimental data and bioinformatic analyses now available, numerous examples of organisms missing enzymes for this pathway are known. Many represent infectious pathogenic species, like intracellular pathogens from the genera *Chlamydia*, *Rickettsia*, and *Mycoplasma*, and parasitic protozoans, e.g., *Trypanosoma*. As purine salvage from the environment is always energetically favorable when compared with de novo synthesis (Brault and Terjung 2001; An et al. 2008), it is not surprising that organisms living in purine-rich environments, like *H. pylori* located in the gastric mucosa, evolved a purine salvage pathway to meet their purine needs. Given the importance of purine production and its direct effect on viability of all cells, targeting enzymes of the purine salvage pathways of pathogenic organisms, for whom this metabolic route is the only source of purine nucleotides, seems promising in the search for drugs with a new mechanism of action, and it has become a heavily investigated field of research. In the present study, we describe inhibitors of two purine salvage pathway enzymes, purine nucleoside phosphorylase (PNP) and adenylosuccinate synthetase (AdSS), from *H. pylori*, with the main goal of establishing these two enzymes as potential molecular targets for new drugs against this pathogen.

Hadacidin (Hada), formycin A (FA), and formycin B (FB) (Fig. 2) inhibit *H. pylori*  26695 strain enzymes in vitro, with the following inhibition constants: 0.19 ± 0.02 μM for hadacidin vs. AdSS (Bubić et al. 2018), as well as 14.0 ± 1.7 μM for FA (Narczyk et al. 2018) and 0.96 ± 0.08 µM for FB vs. PNP (Fig. 6). However, in in vivo experiments, these compounds do not significantly affect the growth of *H. pylori*  26695 and SS1 strains (Table 1).

To see if this observed discrepancy results from a decreased compound penetration into *H. pylori* cells, we checked the cellular uptake of these compounds by the *H. pylori*  26695 strain. Based on the idea of Zhou et al. (2015), a new universal method was developed, which is suitable a priori for checking cellular *H. pylori* uptake of any possible inhibitor, with no need for isotope or fluorescent labelling. In this approach, the cellular uptake is quantified by measuring the reduction of the inhibitor concentration in the external culture medium, and numerous detection methods were shown to be suitable: LC–MS and UV absorption, as well as the inhibition of the target enzyme. The method has been confirmed using metronidazole, an antibiotic used in *H. pylori* therapies (e.g., Min Kim et al., 2020; Olmedo et al. 2020).

Extracellular concentration of the formycins was monitored by LC–MS, as described by Zhou et al. (2015), but it was shown that UV absorbance of these two inhibitors, shifted to a longer wavelength than the cell culture medium absorbance (Fig. 5a), may also be used to obtain results of essentially the same quality (Fig. 7a, b, c). However, this method of detection required a correction for the absorbance of metabolic products released by *H. pylori* cells. Otherwise, an apparent increase of the inhibitor concentration in the extracellular supernatants could be observed when the observation wavelength was shorter than about 300 nm (Fig. 5b, c). When the proper correction was done, the UV absorbance detection method turned out to be accurate and easy to perform. Additionally, it yielded results consistent with those obtained by the LC–MS method (Fig. 7a, b, c).

However, hadacidin is too small and too volatile to be detected by MS, and it exhibits UV absorption that overlaps with the UV absorption of the cell culture medium. Thus, neither of these detection methods were applicable. For this particular compound, we developed a new method of detection, based on the fact that hadacidin is an inhibitor of the AdSS enzyme. Therefore, the cellular uptake of hadacidin was quantified by measuring the remaining inhibition of AdSS by supernatant from the accumulation test (Fig. 8a). While the LC–MS and UV absorbance detection methods work quite well for the formycins, we have shown that inhibition of the target enzyme, in this case PNP, may be used for these compounds as well.

The relative value of the correction for UV absorption by *H. pylori* metabolites is larger as the starting inhibitor concentration is lower; hence, the UV absorption detection method works better for higher inhibitor concentrations. In contrast, the third detection method, inhibition of the target enzyme activity, is more suitable for low inhibitor concentration. Hence, these two methods complement each other.

Detection by the analysis of the inhibition of the target enzyme activity is prone to higher errors than two other methods. One reason for that is that the concentration of the inhibitor in the supernatant is inversely proportional for the measured parameter, enzyme activity (see Eq. (4)), while in two other methods, it is directly proportional (to absorbance in a chosen observation wavelength in the UV absorption detection and to area under the peak in the LC–MS detection). Nevertheless, the standard error for inhibition of the target enzyme activity can be reduced by increasing the number of replicates, and this may be achieved by adapting the method for a microplate reader. One of the most prominent features of this inhibition of target enzyme activity strategy, which provides superiority over other methods, is that it may always be used when we know at least one target enzyme of the tested inhibitor. It may be an enzyme from a different organism than the organism for which the accumulation test is carried out, and it may even be an enzyme exhibiting a different activity that is still a target for the tested inhibitor. In our case, an enzyme other than PNP that is also inhibited by formycins could possibly be used to quantify cellular uptake of formycins, as PNP was found to be inhibited just by the metabolite products expelled by *H. pylori* cells to the incubation medium.

Our results (Table 2) show that hadacidin at 35 µM does not penetrate into *H. pylori* cells (cellular uptake of 4.9 ± 4.1 µM), while formycin B at 350 µM and 35 µM exhibits minimal uptake, as the extracellular starting concentration of 350 µM was lowered by 7.1 ± 2.5 μM and 2.5 ± 0.5 μM (Table 2, data from the LC–MS detection). For formycin A at 35 µM and 350 µM, some modest uptake is observed, as the corresponding decrease in the starting concentration was 15.9 ± 0.5 μM and 29.5 ± 5.3 μM, respectively. These differences could be in some extent explained by the differences in hydrophobic/lipophilic properties of these compounds (Table 3), as some sort of correlation between these parameters may be noticed. At pH 7 only hadacidin is charged and has the lowest partition coefficient (− 4.06), while partition coefficients for both formycins are about two orders of magnitude higher, − 2.06 and − 1.90 for formycin B and A, respectively (Table 3). Indeed, all three compounds very poorly penetrate into *H. pylori* cells, with hadacidin showing the worst uptake, if any, and formycin A — the best from the three studied compounds. Moreover, good uptake of metronidazole correlates well with its relatively high log P =  − 0.47 (Table 3). These data indicate that difference between in vitro and in vivo action of the three analyzed PNP/AdSS inhibitors is indeed the result of their poor uptake by *H. pylori* cells, caused by their small partition between the lipid and aqueous phases. Therefore, one of possible approaches to enhance the activity of these inhibitors is to combine them with compounds taken up through the bacterial inner membrane. This strategy requires further exploration.

**Table 3:** Partition coefficients of the neutral and ionized forms, and pK_a_ values of the tested compounds, and of metronidazole used as a control in the cellular uptake studies. Partition coefficients of the ionic forms observed at pH 7 are marked bold

Inhibitor	logP^a^	logD^a,b^	Ionic form at pH 7	pK_a_^c^
Formycin A	** − 1.90**	** − **4.64	Neutral	4.6; 9.7
Formycin B	** − 2.06**	** − **4.63	Neutral	0.9; 8.6
Hadacidin	** − **2.26	** − 4.06**	Anion	3.5; 9.4
Metronidazole	** − 0.47**	n.d	Neutral	2.38

^a^Coefficients logP and logD were calculated using the Molinspiration Cheminformatics website http://www.molinspiration.com

^b^logD refers to the water:octanol partition coefficient of the ionized form, namely N(4) protonated formycin A, N(6) deprotonated formycin B and hadacidin with deprotonated carboxyl group; formycin ring numbering is shown in Fig. 2

^c^pK_a_ values and protonation sites for formycins from Giziewicz and Shugar (1977); Wierzchowski and Shugar (1982); Bzowska et al. (1992), for hadacidin from Gottlieb and Shaw (2013) and for metronidazole from Hellgren et al. (1995)

The fractional inhibitory concentration index (FICI) was also measured to check if a combination of two inhibitors caused a synergistic or additive effect (Pillai et al., 2005). In the case of formycin A and B vs. SS1 strain, the FICI index points to an additive effect. Both compounds are inhibitors of PNP; hence, they compete for the same enzyme. Thus, this result may indicate that in *H. pylori* there is another enzymatic target for one (or both) of these compounds. This does not seem improbable as both are analogues of naturally occurring purine nucleosides (Fig. 2), adenosine and inosine, respectively.

Although the three inhibitors examined in this study are far from being useful in clinic, the results obtained are very encouraging. The effects observed in vivo are very modest due to poor penetration of these compounds to the *H. pylori* cells, but this obstacle could probably be overcome using a suitable drug delivery system (e.g., see Zhang et al. (2019). This new method to measure cell penetration of inhibitors, developed by us, will be capable of determining *H. pylori* cellular uptake of every future inhibitor designed as a potential drug against this pathogen. It may, in principle, be extended to cellular uptake by any cell type. We think that the detection method, based on the inhibition of the target enzyme of the studied compound, is especially promising when applied in antibiotic drug discovery, as a versatile solution to study the uptake measurement.

Returning to our research, it seems there is an urgent need to search for other PNP and AdSS inhibitors besides those analyzed here, to better judge the usefulness of both enzymes as targets for new drugs against *H. pylori*. Moreover, the additive effect of formycin A and B indicates that there is a need to analyze whether other enzymes of the purine salvage pathway could also serve as targets. The fact that the Δ*deoD* mutant is incapable of growth on the purine base adenine hints at potential existence of an as-yet unknown adenosine deaminase in *H. pylori* (Liechti and Goldberg 2012).

A good starting point for designing inhibitors of PNP and AdSS from *H. pylori* is the solved X-ray structure of the PNP complex with formycin A (Narczyk et al. 2018), and the recently solved structure of AdSS in a complex with hadacidin (to be published; PDB 6ZXQ). Such studies are currently in progress in our laboratories.

## Data Availability

Data and materials will be made available on reasonable request (please send to AB).
